# Proposal and Numerical Analysis of Organic/Sb_2_Se_3_ All-Thin-Film Tandem Solar Cell

**DOI:** 10.3390/polym15112578

**Published:** 2023-06-05

**Authors:** Tarek I. Alanazi, Abdulaziz Alanazi, Ezzeddine Touti, Ahmed M. Agwa, Habib Kraiem, Mohana Alanazi, Abdulrahman M. Alanazi, Mona El Sabbagh

**Affiliations:** 1 Department of Physics, College of Science, Northern Border University, Arar 73222, Saudi Arabia; 2 Department of Electrical Engineering, College of Engineering, Northern Border University, Arar 73222, Saudi Arabia; af.alanazi@nbu.edu.sa (A.A.); esseddine.touti@nbu.edu.sa (E.T.); ahmad.agua@nbu.edu.sa (A.M.A.); habib.kraiem@yahoo.fr (H.K.); st201903004@stu.nbu.edu.sa (A.M.A.); 3 Electrical Engineering Department, Laboratory of Industrial Systems Engineering and Renewable Energies (LISIER), University of Tunis, Tunis 1008, Tunisia; 4 Department of Electrical Engineering, Faculty of Engineering, Al-Azhar University, Cairo 11651, Egypt; 5 Processes, Energy, Environment and Electrical Systems, National Engineering School of Gabes, University of Gabes, Gabes 6029, Tunisia; 6 Department of Electrical Engineering, College of Engineering, Jouf University, Sakaka 72388, Saudi Arabia; msanazi@ju.edu.sa; 7 Engineering Physics and Mathematics Department, Faculty of Engineering, Ain Shams University, Cairo 11535, Egypt; mona.mohammed@eng.asu.edu.eg

**Keywords:** thin film, tandem solar cell, Sb_2_Se_3_, organic, current matching condition, Silvaco TCAD

## Abstract

The low bandgap antimony selenide (Sb_2_Se_3_) and wide bandgap organic solar cell (OSC) can be considered suitable bottom and top subcells for use in tandem solar cells. Some properties of these complementary candidates are their non-toxicity and cost-affordability. In this current simulation study, a two-terminal organic/Sb_2_Se_3_ thin-film tandem is proposed and designed through TCAD device simulations. To validate the device simulator platform, two solar cells were selected for tandem design, and their experimental data were chosen for calibrating the models and parameters utilized in the simulations. The initial OSC has an active blend layer, whose optical bandgap is 1.72 eV, while the initial Sb_2_Se_3_ cell has a bandgap energy of 1.23 eV. The structures of the initial standalone top and bottom cells are ITO/PEDOT:PSS/DR3TSBDT:PC_71_BM/PFN/Al, and FTO/CdS/Sb_2_Se_3_/Spiro-OMeTAD/Au, while the recorded efficiencies of these individual cells are about 9.45% and 7.89%, respectively. The selected OSC employs polymer-based carrier transport layers, specifically PEDOT:PSS, an inherently conductive polymer, as an HTL, and PFN, a semiconducting polymer, as an ETL. The simulation is performed on the connected initial cells for two cases. The first case is for inverted (p-i-n)/(p-i-n) cells and the second is for the conventional (n-i-p)/(n-i-p) configuration. Both tandems are investigated in terms of the most important layer materials and parameters. After designing the current matching condition, the tandem PCEs are boosted to 21.52% and 19.14% for the inverted and conventional tandem cells, respectively. All TCAD device simulations are made by employing the Atlas device simulator given an illumination of AM1.5G (100 mW/cm^2^). This present study can offer design principles and valuable suggestions for eco-friendly solar cells made entirely of thin films, which can achieve flexibility for prospective use in wearable electronics.

## 1. Introduction

Solar energy resources have become practical substitutes for conventional fossil fuels because of their sustainability and renewability [1]. To develop photovoltaic (PV) solar cells, it is necessary to improve their efficiency to be able to compete in the industrial energy sector [2]. The power conversion efficiency (PCE) of a solar cell device can be effectively boosted through the fabrication of tandem cells that have several absorption photoactive layers with complementary bandgaps [3]. A two-terminal (2T) tandem configuration, which is the best choice for cost-effective applications, integrates a wide bandgap absorber as a front subcell and another narrow bandgap absorber as a bottom subcell. The front and bottom subcells are electrically tied through an interlayer. This interconnection is identified as the recombination layer and can simply be an ultrathin film of a metal such as Ag [4]. Notably, a high PCE can be obtained in a tandem cell as a consequence of incorporating a broader part of the solar spectrum thanks to the complementary bandgaps used. Additionally, the thermalization losses of photon energy can be alleviated. It was revealed that a 2T monolithic tandem that has a top subcell bandgap energy (*E_g_*) of 1.7 eV along with a 1.12 eV rear subcell can achieve a theoretical PCE of up to 40% [5].

Tandem solar cells have been employed for a broad variety of absorber materials. The leading competitor in PV technologies is Si, which is also one of the most widely utilized bottom cells in tandem devices, alongside its use as a single junction cell, as Si has a narrow *E_g_* of about 1.12 eV, while its PCE has a high record of 26.8% [6]. However, Si-based cells incorporate energy-rigorous manufacturing processes which lead to relatively high production costs [7]. In addition, the thickness of Si wafers is relatively high, which prevents them from being applied in flexible applications such as wearable electronics. Although there are some research studies aimed at decreasing the wafer thickness and processing cost of Si solar cells [8,9,10,11,12,13], the PCEs of such Si-based cells are still far behind the conventional crystalline Si solar cells.

Among the numerous types of solar cell devices, other than Si, thin-film solar cells (TFSCs), which represent the second generation of PV technology, have been recognized as potential and promising contenders to be used in PV applications owing to their low material cost, low-temperature processes, flexibility, and compatibility with mass production [14]. Therefore, TFSCs can offer a favorable approach to achieving elevated PCEs while maintaining low costs a priory. So far, numerous rear subcells with narrow bandgap energies have been proposed, including, for instance, CIGS [15], organic solar cells (OSCs) [16], and perovskite solar cells (PSCs) [17]. While CIGS has achieved an extraordinary PCE of 23.3% [18], issues remain concerning the scarcity of the constituting materials of the CIGS, namely, Te, In, and Ga [19]. Additionally, low bandgap PSCs are usually based on Sn, which is less efficient in addition to its worse stability than the lead-based perovskite [20]. Thus, there is room to investigate and allow the introduction of other photoactive materials to be employed as alternative options for the rear subcell.

One of the most promising cheap and non-toxic PV materials is the binary Sb_2_Se_3_ semiconductor, which has a lot of other advantageous features. Firstly, Sb_2_Se_3_ consists of earth-abundant elements that show superior stability. Secondly, the electrical properties of Sb_2_Se_3_ are suitable for bottom subcells in tandem devices as its direct bandgap energy is in the order of 1.2 eV, while its carrier mobility is relatively high in the order of 10 cm^2^/V.s [21]. Moreover, the optical characteristics are appropriate as it has large extinction and absorption coefficients [21]. Based on these interesting properties, Sb_2_Se_3_ has been developed as a favorable PV material and has received growing research appeal [22,23,24,25]. After a few years of development, the PCE of Sb_2_Se_3_-based solar cells has gradually expanded [26]. Recently, a newly developed additive-assisted chemical bath deposition (CBD) technique has been used to produce high-quality Sb_2_Se_3_ films, resulting in the highest recorded PCE of 10.57% for Sb_2_Se_3_-based solar cells [27]. Thus, the thin-film Sb_2_Se_3_ material can be a promising alternative to Si as a bottom subcell thanks to its growing research interest and rapid boosting of its PCE. Eventually, a study has recently explored Sb_2_Se_3_ (with a reported *E_g_* of 1.22 eV) as a bottom subcell while incorporating Sb_2_S_3_ (with a reported *E_g_* of 1.74 eV) as a top cell [28]. The mentioned experimental work confirms the suitability of the Sb_2_Se_3_ cell as a rear subcell of the tandem which demonstrates a proof of concept. Moreover, a theoretical study of a triple-junction antimony chalcogenides solar cell revealed a PCE of 32.98% when designing the tandem based on Sb_2_S_3_/Sb_2_(S_0.7_Se_0.3_)_3_/Sb_2_Se_3_ stack configuration [29].

When concerning suitable top subcells, on the other hand, the OSC can be a superb target. OSCs have attracted great research attention because of their various advantages, which include ease of manufacturing, low weight, and cheap processing cost. A maximum certified achieved PCE of a single-junction OSC is above 18% [30]. Tandem OSCs have been extensively reported in the literature. Some of these tandems are constructed from polymer donor materials and fullerene acceptor materials with a PCE of 11.6%, while others are created from polymer small-molecule donor and fullerene acceptor materials with a PCE of 12.7% [31,32]. X. Che et al. fabricated a tandem device using a norfullerene acceptor in the back subcell and a small molecule material in the top cell resulting in a PCE of 15% [33]. Further, recent studies consider small-molecule-based cells because they usually have a relatively higher open circuit voltage than polymer-based counterparts [34]. This characteristic, specifically, is extremely beneficial in developing tandem solar cells. Furthermore, the purity of small-molecule materials is high, and the control of thin-film thickness is manageable, alongside their ability to be coated in a large area, implying the ease of their commercialization [35]. In [36], an optimized tandem device that is based on a small-molecule organic front subcell with an optical *E_g_* of 1.72 eV has been fabricated, and a PCE of 12.50% has been attained. Moreover, a PCE of 16.40% has been accomplished for a tandem OSC comprised of a small-molecule acceptor material *m*-DTC-2F with a bandgap energy of 1.61 eV used in the front subcell [37].

Based on the previous discussion, Sb_2_Se_3_ and organic materials can have complementary absorption behavior upon a proper design of the organic blend. These photoactive films are deemed eco-friendly solar cells, in addition to their low processing cost. Further, because of the nature of Sb_2_Se_3_ and organic solar cells, flexible cell devices with a superior power-to-weight ratio can be produced. Therefore, the current simulation study introduces a tandem device that integrates organic (with a bandgap of 1.72 eV) and Sb_2_Se_3_ (1.23 eV) absorber materials to be used for the front and bottom cells, respectively. First, the simulation model and the physical and technological parameters are validated through the simulation of two standalone organic and Sb_2_Se_3_-based cells that were previously fabricated [36,38]. The calibration process is provided by comparing the simulation results against measurements. Moreover, both inverted (p-i-n)/(p-i-n) and conventional (n-i-p)/(n-i-p) tandem structures are investigated to compare them and determine which configuration is more promising. For both cases, the initial arrangements are simulated. Then, the optimization of the main layer materials is carried out to decide which the most dominant influence is on the tandem cell performance. Followed by this step, the current matching condition is applied for both types to achieve the optimum efficiency.

By introducing the organic solar cell with an active blend layer (DR3TSBDT:PC71BM) and the use of Sb_2_Se_3_ as the bottom cell, we present a unique approach that offers several advantages, including the potential for low-cost production due to the utilization of organic materials and the flexibility provided by the organic layer. The integration of Sb_2_Se_3_ as the bottom cell adds to the novelty of our work, as Sb_2_Se_3_ has recently gained attention for its desirable optoelectronic properties and potential applications in photovoltaics in addition to its low cost and flexibility. By investigating both the n-i-p and p-i-n structures for the tandem configuration, we further expand the scope of our study and provide valuable insights into the performance and potential advantages of each structure. The presented thorough analysis contributes to the understanding of the device’s physics and the optimization strategies for this unique combination.

## 2. Simulation Technique and Device Configurations

### 2.1. Simulation Environment

In this work, 2-D TCAD device simulations are accomplished by using the Silvaco Atlas simulator. The operation of the Atlas simulator is built on resolving electron and hole transport equations self-consistently, alongside employing Poisson’s equation at each predefined mesh. All relevant physical models, which are crucial for solar cell devices, are included in the simulations. Regarding recombination, Shockley–Read–Hall (SRH) and Auger mechanisms are enabled. Optical recombination is also incorporated. Further, Fermi statistics are used to describe carrier statistics, especially in high-doped regions. To characterize the hopping phenomenon that occurs through the boundary between the two subcells in the tandem device, a lumped resistance is added to model the interlayer. In addition, a concentration-dependent mobility model is invoked to include the effect of high doping on carrier mobilities.

The flow of the Atlas simulator’s working steps is summarized as follows: First, 2-D Luminous provides input spectra for AM1.5G conditions by utilizing a BEAM statement. To accurately model light propagation, the variation of the extinction coefficient of the materials comprising the solar cell versus wavelength should be carefully provided. Next, the 2-D ray tracing method or 1-D transfer matrix method is selected to calculate the photogeneration rate along the device. Then, the photogeneration rate is integrated in the electron and hole continuity equations where a new carrier concentration is determined. The electrical simulation is then conducted by solving the drift-diffusion model along with Poisson’s equation. The process is iteratively carried out to obtain the final carrier concentration and, finally, the terminal current density, from which the basic cell performance parameters can be easily extracted. More details about the Atlas simulator can be found in the Atlas manual from Silvaco [39].

### 2.2. Subcell Device Structures

**Figure 1:**
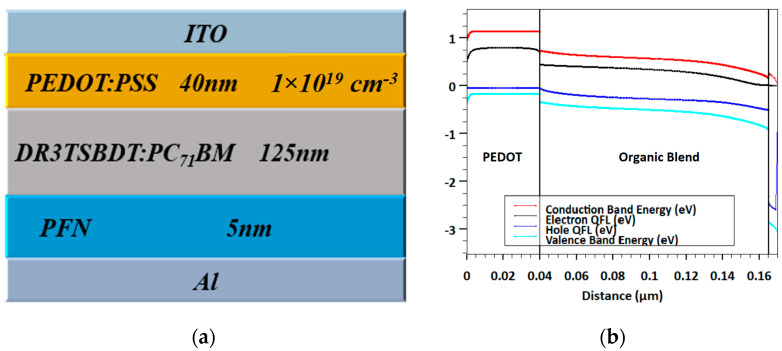
(**a**) Subcell device structure including the various layers of organic solar cell and (**b**) corresponding energy band profile at short circuit.

The top cell is an OSC, which is characterized by a p-i-n heterojunction device whose structure is represented as demonstrated in Figure 1a. This structure is based on a practical study in which the layers are as follows: A transparent contact (ITO) (that has a work function of 4.6 eV) is followed by a hole transport layer (HTL) of the inherently conductive PEDOT:PSS polymer whose thickness is 40 nm. Next, the active layer consists of DR3TSBDT:PC_71_BM, whose optical bandgap is about 1.72 eV and has a thickness of 125 nm. The electron transport layer (ETL) is a semiconducting PFN polymer with a thickness of 5 nm. The back contact is formed from Al whose work function is taken as 3.9 eV. All technological and physical factors of the cell are extracted from REF [36]. The energy band profile of the cell is shown in Figure 1b, in which the condition of the short circuit under illumination has been taken.

**Figure 2:**
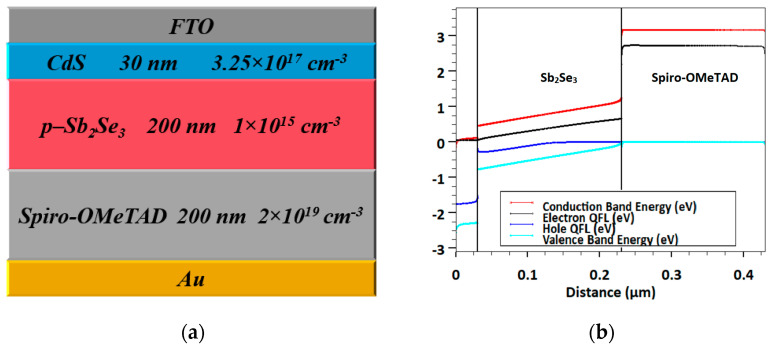
(**a**) Subcell device structure including the various layers of Sb_2_Se_3_ solar cell and (**b**) corresponding energy band profile at short circuit.

Conversely, the rear cell is based on Sb_2_Se_3_, which is sandwiched between CdS and Spiro-OMeTAD that act as ETL and HTL, respectively. The construction of this device is an n-i-p heterojunction as displayed in Figure 2a. Figure 2b demonstrates the energy band profile under illumination and short circuit operation. The top and back contacts are established from FTO and Au, respectively. The thicknesses of CdS, Sb_2_Se_3_, and Spiro-OMeTAD are 30 nm, 200 nm, and 200 nm, respectively. The work functions of FTO and Au contacts are kept at 4 eV and 5.1 eV, respectively [40]. The main factors are extracted after a previously fabricated cell that has the same layers’ configuration [38].

Table 1 presents the key physical and geometrical factors of all layers of the top and bottom cells. The conduction band minimum (CBM) along with the valence band maximum (VBM) are given, from which one can calculate the electron affinity and electrical bandgaps of the distinct layers. It should be pointed out here that the optical bandgap of the organic blend material is different from that of the electrical bandgap. The electrical bandgap of the organic blend is computed from the difference between the CBM and VBM levels, while the optical bandgap is determined from the cutoff wavelength of the absorption coefficient of the blend. Furthermore, Table S1 (see Supplementary Materials) addresses the defect parameters used through all simulations for Sb_2_Se_3_ [23,25,38]. Regarding the OSC, as there is no available date for the defects, a donor trap level whose energy level locates at 0.5 eV is assumed. The hole and the electron capture cross sections are taken as 1 × 10^−17^ cm^2^, while the defect trap density is fitted at 5 × 10^12^ cm^−3^ to obtain the best fit of experimental data. Additionally, the optical properties in terms of extinction coefficients are taken from the literature [36,38].

### 2.3. Calibration of Modeling Technique

**Figure 3:**
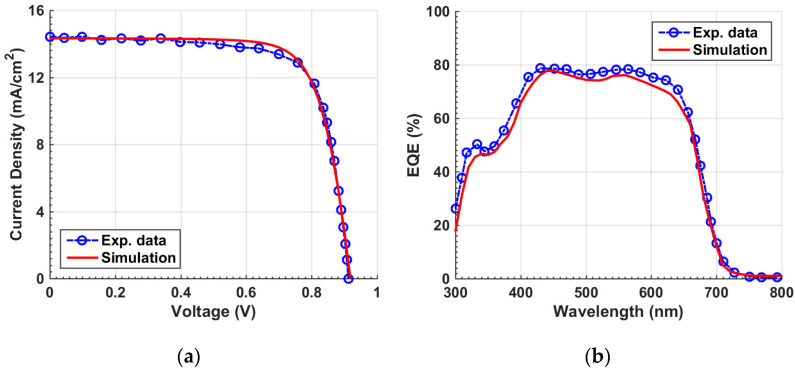
OSC calibration: simulation vs. measured data. (**a**) Illuminated *J–V* curves and (**b**) EQE spectra.

**Figure 4:**
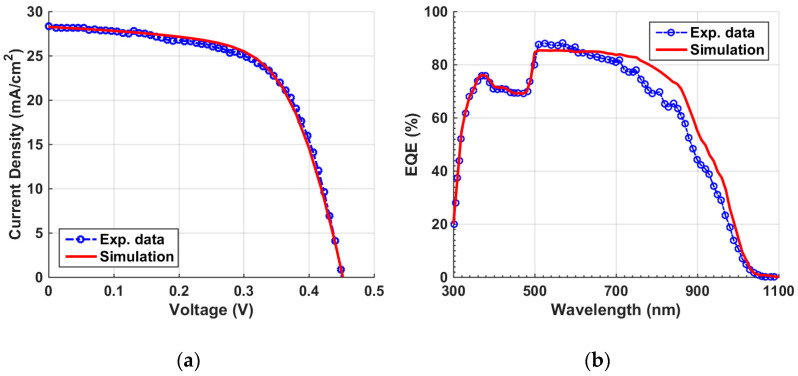
Sb_2_Se_3_ cell calibration: simulation vs. measured data. (**a**) Illuminated *J–V* curves and (**b**) *EQE* spectra.

When applying the given parameters listed in Table 1 and Table S1 for the individual organic and Sb_2_Se_3_ cells, the optoelectronic simulation output regarding the current density–voltage (*J–V*) characteristic curves, found under the illumination condition, and the external quantum efficiency (EQE) spectra are displayed in Figure 3 and Figure 4, respectively. Both simulation and experimental results are shown in the figures. According to this calibration step, the PV performance measures of the cells are demonstrated in Table 2. The experimental PV parameters reported in table are derived from the previously fabricated organic and Sb_2_Se_3_ solar cells as mentioned earlier [36,38]. The simulated *J–V*, EQE spectra, and PV key parameters are nicely reproduced by simulation in both types of cells, implying the validation of the modeling approach implemented in the Atlas simulator to handle these TFSCs.

### 2.4. Proposed Tandem Structures

**Figure 5:**
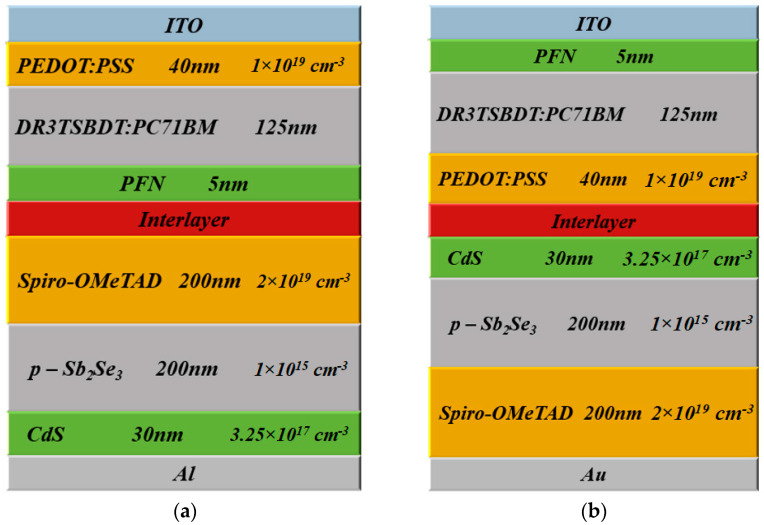
Tandem device structure (**a**) inverted p-i-n and (**b**) conventional n-i-p.

In this work, both types of conventional and inverted tandem devices will be investigated to highlight the possible ways of boosting tandem performance. Figure 5a and Figure 5b represent the (p-i-n)/(p-i-n) inverted structure and (n-i-p)/(p-i-p) conventional structures, respectively. Regarding the (p-i-n)/(p-i-n) arrangement, the p-doped HTL is confronting the illumination, while, in the (n-i-p)/(n-i-p) configuration, the n-doped ETL is facing the illumination. In the following section, the simulation results of the two cases are presented including the optimization of the two designed tandem devices.

## 3. Results and Discussion

In this part, the simulation results of both inverted and conventional tandem structures will be presented. In this first part of the section, the focus is on the inverted tandem in the (p-i-n)/(p-i-n) architecture. In the second part, the conventional (n-i-p)/(n-i-p) tandem cell is to be explored. In both cases, the initial arrangements are simulated. Then, the optimization of the main transport layer materials is carried out to determine the most dominant effect on the tandem performance. Next, the influence of thickness of the top and rear absorbers is explored. Followed by this step, the current matching situation is applied for both types to obtain the optimum PCE.

### 3.1. Inverted (p-i-n)/(p-i-n) Tandem Design

**Figure 6:**
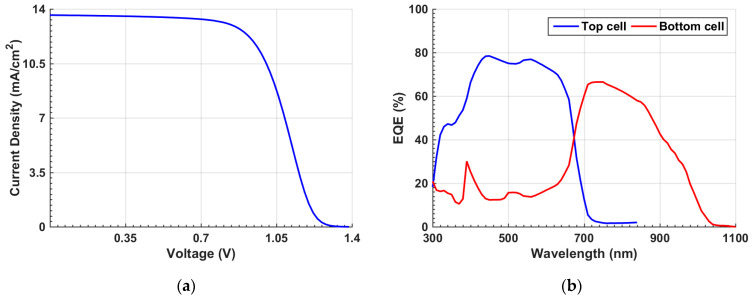
Initial inverted tandem characteristics (**a**) *J–V* curves under illumination and (**b**) EQE curves.

The inverted configuration is shown in Figure 5a. Figure 6a displays the *J–V* characteristic curve of the tandem device. The output parameters reveal a *V_oc_* of 1.3 V, *J_sc_* of 13.61 mA/cm^2^, FF of just 60.21%, and a PCE of 11.28%. Additionally, the EQE curve versus wavelength is exhibited for the front and bottom subcells in Figure 6b. The top subcell demonstrates a maximum EQE around 400 nm, while the bottom subcell demonstrates a maximum EQE at about 730 nm. The short wavelength zone nearly below 680 nm is primarily absorbed through the top subcell, while the bottom subcell is responsible for the absorption in the wavelength range among 680 nm and 1100 nm as revealed in Figure 6b. Although there was a proper complementary absorption in the tandem, the S-curve phenomenon indicated in Figure 6a indicates that the initial tandem device should be optimized to control the band misalignment that is responsible for the kink effect.

#### 3.1.1. Optimization of Transport Layers Materials

In order to alleviate the occurrence of the kink effect, a proper design that results in band alignment between the distinct tandem layers have to be performed. This can be conducted by varying the ETL and/or ETL materials to obtain appropriate values of conduction and valence band offsets that assist the transport of the photoexcited electrons and holes towards the corresponding contacts. In this regard, two important parameters are defined, namely, the conduction band offset (CBO) and the valence band offset (VBO). The CBO at the ETL/absorber interface is computed from the difference between the electron affinities of the absorber and ETL (CBO = *χ*_abs_ − *χ*_ETL_). In contrast, the VBO at the absorber/HTL interface is computed as VBO = (χ_HTL_ + *E_g_*_HTL_) − (χ_abs_ + *E_g,_*_abs_). The optimum CBO was found to be in the range 0 to 0.3 eV, while that of the VBO was found in the range 0 to 0.2 eV [46].

**Figure 7:**
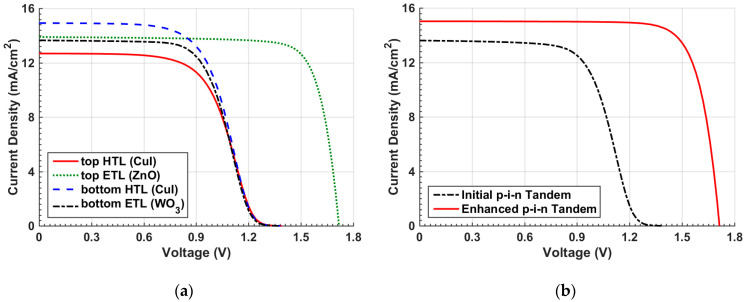
(**a**) Comparison of *J–V* behavior between various cases of optimization of inverted tandem design and (**b**) *J–V* curves of initial versus optimized transport layers tandem.

To control the band alignment, the top HTL is replaced by CuI to alter the VBO. The ETL material of the top subcell is also replaced by ZnO to engineer the CBO. Next, the HTL and ETL materials of the bottom Sb_2_Se_3_ subcell are replaced by CuI and WO_3_, respectively. The chosen materials as HTL or ETL candidates are selected according to previous experimental studies concerning OSCs or Sb_2_Se_3_ cells [47,48,49,50,51,52]. The main material parameters for CuI, ZnO, and WO_3_ are given in Table S1 in the Supplementary Materials [44,45,53]. In each step of these simulations, only one material is individually changed to inspect its own effect aside any other change. Table 3 addresses the various optimization steps along with the initial results to give a comparative picture. Moreover, the relative change in efficiency (Δ*ξ*) is specified, which measures the enhancement in the cell performance. Further, a comparison of the various optimization steps regarding the distinctive transport layer materials is shown in Figure 7a in terms of the illuminated *J–V* curves. As can be depicted in Table 3, replacing the HTL of the OSC to CuI is not recommended, although its mobility is higher than PEDOT:PSS. This is due to the inappropriate value of the barrier height between the front contact and the VBM of the HTL as its value is about 0.6 eV, while its value in the case of PEDOT:PSS is 0.3 eV. This high barrier in the CuI case lowers the current, as shown in Figure 7a.

On the other hand, when replacing the top ETL with ZnO, a considerable improvement in the cell performance, reflected on a higher PCE, can be noticed. This can be attributed to the doping effect of the ZnO layer as ZnO can be n-doped while PFN is assumed undoped in the simulation. The CBO in the case of ZnO is −0.1 eV in comparison to the initial CBO value of 0.1 eV. So, the major impact does not come from the CBO variation as the values of the CBOs are near each other’s. Now, when changing the transport layer materials of the bottom subcell, it can clearly be observed that the impact of bottom HTL CuI gives a slight enhancement, while the bottom ETL has an irrelevant effect as it is the last layer of the structure. Thus, the enhancement of the overall tandem performance can be accomplished by changing the ETL and HTL of the top and bottom subcells, respectively. When applying both modifications, a PCE of 20.45% can be achieved. The other PV parameters are listed in Table 3. Moreover, a plot of *J–V* of the optimized layer materials and the initial tandem cell design are displayed in Figure 7b. This figure obviously demonstrates the disappearance of the kink effect from the optimized case when compared to the initial case.

#### 3.1.2. Optimization of Top and Bottom Absorbers Thickness

**Figure 8:**
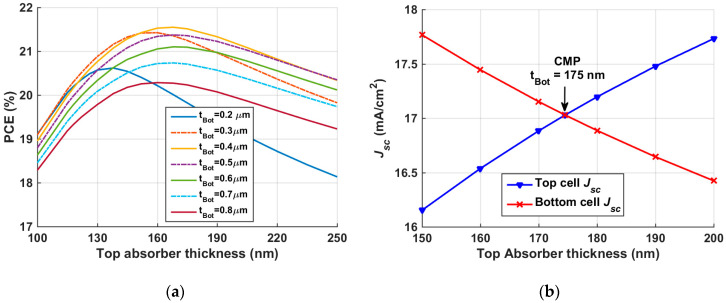
(**a**) p-i-n tandem PCE dependency on the thickness of front and back absorber films; (**b**) *J_sc_* of top and bottom subcells against top cell absorber thickness.

In order to obtain the maximum achievable PCE from the tandem, both top and bottom absorbers thicknesses are concurrently varied. The range of the top absorber thickness is taken from 100 nm to 250 nm, while the thickness of the bottom absorber is varied in the range 0.2 to 0.8 µm. For a given value of the bottom absorber thickness, increasing the top absorber thickness improves the PCE up to a certain value, after which the PCE declines as can be depicted in Figure 8a. Contour plots of the other set of PV parameters are shown in Figure S1 in the Supplementary Materials. An optimum value of the bottom absorber thickness can be taken as 0.4 µm. A top absorber thickness in the order of 170 nm can be chosen to achieve the maximum PCE according to the results in Figure 8a; however, to choose a precise value of the top thickness and give more physical insight into the trend of the current through the two subcells, an optimization routine is carried out to determine the matching current mode.

For a 2-T connected tandem device, it is remarkably crucial to achieve balanced currents through subcells. Therefore, to accurately match the short-circuit currents produced from the top and bottom subcells, the top absorber thickness is changed from 150 nm to 200 nm, given a bottom absorber thickness of 0.4 µm as indicated herein, and the resulting variation in top and bottom *J_sc_* is illustrated in Figure 8b. The figure signifies that the top *J_sc_* increases while the bottom *J_sc_* decreases upon increasing the top absorber thickness. This trend can be ascribed to the fact that the strong absorption on thick top absorber film causes less absorption in the bottom subcell and vice versa. Notably, the intersection of both *J_sc_* curves corresponds to the matching point which occurs at a top absorber thickness of 175 nm.

**Figure 9:**
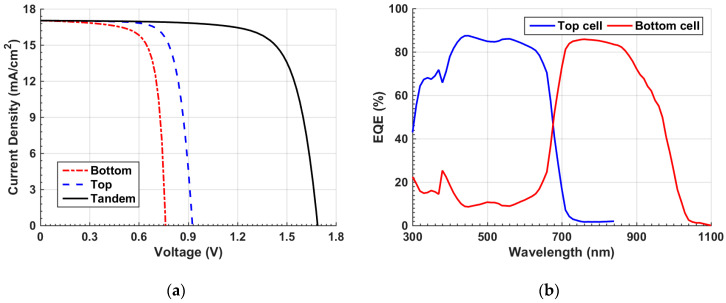
Characteristics under current matching point (**a**) *J–V* of tandem, top, and bottom subcells and (**b**) *EQE* curves of front and rear subcells.

Finally, *J–V* and EQE characteristic curves of the top and bottom subcells and the overall tandem under illumination are offered in Figure 9a and Figure 9b, respectively. Figure 9a clearly shows the confirmation of the current matching condition between the subcells. Furthermore, Table 4 gives a summary of the PV parameters. The *J_sc_* of top, bottom, and tandem are equal (17.03 mA/cm^2^). The *V_oc_* of the tandem (1.68 V) is the sum of the individual subcell open-circuit voltages (0.92 V and 0.76 V). Moreover, the PCE of the tandem cell is boosted to 21.52%.

### 3.2. Conventional (n-i-p)/(n-i-p) Tandem Design

**Figure 10:**
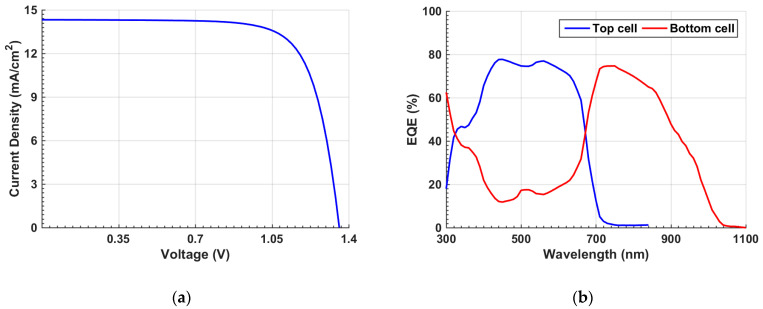
Initial n-i-p tandem characteristics (**a**) *J–V* curves under illumination and (**b**) EQE curves.

The same steps applied for the inverted cell are also employed here to provide design steps for the conventional n-i-p configuration which is exhibited in Figure 5b. First, Figure 10a provides the *J–V* of the tandem upon applying the initial parameters. The PV parameters reveal a *V_oc_* of 1.36 V, *J_sc_* of 14.32 mA/cm^2^, FF of 74.65%, and a high PCE of 14.50%. These tandem metrics are higher than those encountered in the inverted tandem, implying a proper design of the p-i-n configuration over the inverted one. This is also clear when looking at the *J–V* characteristics, which exhibit no S-curve behavior. Moreover, the EQE spectra are presented in Figure 10b for the top and bottom subcells.

#### 3.2.1. Optimization of Transport Layers Materials

**Figure 11:**
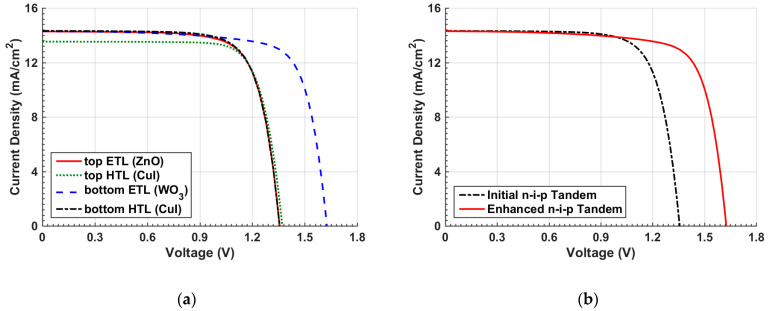
(**a**) Comparison of *J–V* behavior between various cases of optimization of n-i-p tandem design and (**b**) *J–V* curves of initial versus optimized transport layers tandem.

Next, some ETL and ETL materials are varied to optimize the tandem performance. In this context, the top ETL is replaced by ZnO, while the top HTL is replaced by CuI. Both changes have a minor effect of decreasing the performance, as indicated in Table 5, which summarizes the output parameters of the various optimization steps along with the initial results. This necessitates the appropriate design of the organic cell in the conventional n-i-p device configuration. On the other hand, the bottom ETL has the strongest impact amongst the optimization steps. Adjusting the ETL of the bottom subcell at WO_3_ boosts the PCE by **Δ*ξ*** = 21.17%, as specified in Table 5. The enhancement when replacing CdS with WO_3_ can be explained based on the corresponding values of the CBO. In the case of CdS, the CBO is −0.33 eV, while it is 0.05 eV in the case of WO_3_. Furthermore, as can be depicted in Table 5, the improvement of the overall tandem performance can be realized by modifying the ETL and HTL of the bottom subcell. When applying these changes, a PCE of 17.58% can be achieved. Moreover, a comparison of the various optimization steps regarding the distinctive transport layer materials is displayed in Figure 11a concerning the illuminated *J–V* curves. Furthermore, the *J–V* curves of the optimized layer materials and the initial n-i-p tandem cell design are exhibited in Figure 11b. It should be pointed out here that the same ETL and HTL are utilized for both design cases of the n-i-p and p-i-n tandem configurations. Of course, more materials can be applied; so, there is still room for further optimization that can be conducted in future work.

#### 3.2.2. Optimization of Top and Bottom Absorbers Thickness

**Figure 12:**
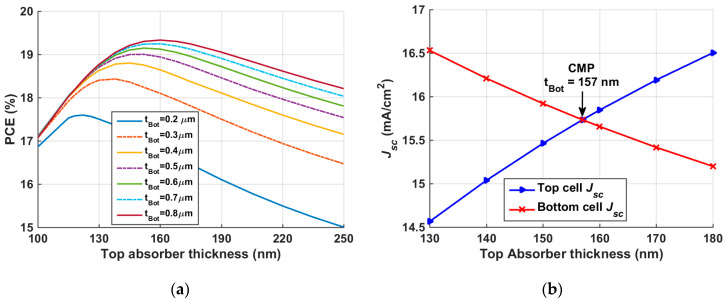
(**a**) Inverted n-i-p tandem efficiency dependency on the thickness of top and rear absorber films; (**b**) *J_sc_* of top and rear subcells against top cell absorber thickness.

Again, to obtain the maximum possible PCE from the tandem, both top and bottom absorbers’ thicknesses are simultaneously varied. The range of the top absorber thickness is taken from 100 nm to 250 nm, while the thickness of the bottom absorber is varied in the range 0.2 to 0.8 µm. It can be inferred from Figure 12a that an optimum value of the bottom absorber thickness can be taken as 0.6 µm, as increasing the bottom thickness beyond this value does not add a lot to the PCE. Moreover, decreasing the bottom absorber thickness is favorable for low cost and easiness of fabrication and flexibility. Contour plots of the other set of PV parameters are shown in Figure S2 in the Supplementary Materials. Next, to accurately match the short-circuit currents produced from the top and bottom subcells, the top absorber thickness is changed from 130 nm to 180 nm, given a bottom absorber thickness of 0.6 µm, and the resulting variation in top and bottom *J_sc_* is illustrated in Figure 12b. The intersection of both *J_sc_* curves corresponds to the matching point which occurs at a top absorber thickness of 157 nm.

**Figure 13:**
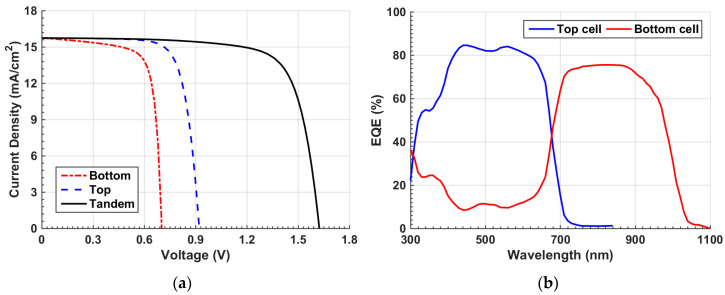
(**a**) *J–V* curves and (**b**) EQE curves of nip tandem-, top-, and rear-cells under current matching point.

Finally, *J–V* and EQE characteristics of top and bottom subcells and the overall tandem under illumination are presented in Figure 13a and Figure 13b, respectively. Figure 13a shows the confirmation of the current matching condition between the subcells. Furthermore, Table 6 gives a summary of the PV parameters. The PCE of the tandem cell is boosted to 19.14%.

### 3.3. Comparison between n-i-p and p-i-n Tandem Designs

**Figure 14:**
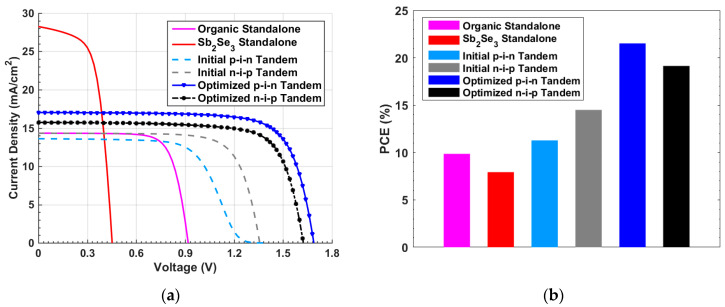
Comparison between key different simulation steps (**a**) *J–V* characteristics and (**b**) PCE of standalone organic and Sb_2_Se_3_, p-i-n and n-i-p initial and optimized tandem cells.

Although inverted-type (p-i-n) solar cell structures have been widely investigated owing to their better stability compared with the conventional structures (n-i-p) [54], more awareness should be given when selecting the HTL material in the inverted-type structure as this material will be deposited on the absorber film. For instance, PEDOT:PSS, as an HTL, is applied as a suspended solution in water, resulting in processing spin-coating issues [55]. The advantage of (n-i-p)/(n-i-p) structure is that utilizing a thin ETL on top of the cell will result in reducing HTL parasitic absorption. In the previous simulations of inverted p-i-n and conventional n-i-p tandem configurations, it was shown that although the initial inverted tandem cell suffers from S-curve shape due to band misalignment, the optimized cell gave a high PCE of 21.52%. Alternatively, while the conventional initial tandem cell showed a better performance compared to the inverted device, the resultant optimized PCE is lower than that of the inverted by 2.38%. These results are summarized in Figure 14, which illustrates the *J–V* curves under illumination (see Figure 14a) and PCE of the various key steps (see Figure 14b).

### 3.4. Comparison between Different Tandem Designs

Finally, we introduce a comparative study between our optimized p-i-n and n-i-p tandems and other thin-film tandem contenders. The comparison is illustrated in Table 7, including both experimental and numerical studies of various tandems. The chosen cells are based on organic, polymer, antimony chalcogenide (Sb_2_X_3_, where X = S, Se, or S*_x_*Se_1−*x*_) or Si subcells. It is evident from the literature that theoretical analysis of tandem devices based on Si has demonstrated high PCE due to the high current capability of the Si bottom cell [56,57]. However, an important finding is that increasing the Si thickness is associated with an increase in PCE which poses a challenge for producing flexible tandems, in addition to the higher cost of Si compared to thin-film technology. In the context of organic/Si tandems, PCEs above 15% have been reported experimentally [58]. However, these tandems lack flexibility as the Si layer thickness is around 300 μm. On the other hand, antimony chalcogenide tandems have been experimentally tested, achieving a PCE of 7.93% for a 4T configuration [28]. Theoretical studies have proposed a triple-junction all-antimony chalcogenide tandem, optimizing different layers in the structure and yielding a high PCE of approximately 33% [29]. Furthermore, there have been extensive designs of organic-based tandem cells, with some experimental efforts reporting efficiencies above 15% [59]. In this context, our simulation of the proposed tandem solar cell demonstrates favorable characteristics, including a high PCE. These results suggest the potential for utilizing our design in all-thin-film tandem solar cells. By highlighting the contrasting findings of Si-based tandems, the limitations of existing tandems in terms of flexibility and thickness, and the promising performance of our proposed design, we underscore the significance and potential of our research in the field of tandem solar cells.

## 4. Conclusions

In this simulation study, a 2T Organic/Sb_2_Se_3_ tandem device has been introduced. The simulation has been performed by utilizing the Atlas device simulator under the illumination of the AM1.5G spectrum. The proposed tandem solar cell utilizes Sb_2_Se_3_ that has a 1.23 eV bandgap as a rear subcell, and an organic blend that has a bandgap of 1.72 eV is incorporated as the front subcell. The work started by calibrating the modeling technique employed in the Atlas simulator. The initial PCEs of previously fabricated organic and Sb_2_Se_3_ single-junction solar cells gave 9.86% and 7.93%, respectively, in good agreement with experimental data. Two proposed structures are then introduced in which the n-i-p and p-i-n tandems were invoked. The initial inverted (p-i-n)/(p-i-n) tandem design showed a kink effect because of the band misalignment. However, after optimizing the tandem, a PCE of 21.52% has been obtained. On the other hand, although the initial conventional (n-i-p)/(n-i-p) tandem design showed a proper band alignment reflected on its *J–V* characteristics, the PCE after optimization was 19.14%, which is less than that of the inverted design. The optimization steps for both cases were the same; we applied different ETL and HTL materials and carried out the current matching condition to obtain the maximum possible PCE.

Overall, the study is based on utilizing small-molecule organic materials which have convenient control of thin-film thickness alongside their ability to be coated in a large area. Moreover, the bottom Sb_2_Se_3_ cell has strong stability behavior and low processing cost. Thus, the presented approach can pave the way for the design of all-thin-film tandem cells, not only those which are flexible and cheap but also those with a competitive PCE. Notably, further optimizations can be applied to boost the performance by investigating more suitable transport materials that can be applied experimentally to offer a realistic simulation that can help direct the experimental studies.

## Figures and Tables

**Table 1:** Main parameters of different sub-cells layers.

Parameters	CdS [38,41,42]	Sb_2_Se_3_ [38,42]	Spiro-OMeTAD [38,43,44]	PEDOT:PSS [36,45]	DR3TSBDT :PC_71_BM [36,45]	PFN [36,45]
Thickness (nm)	30	200	200	40	125	5
VBM (eV)	−6.58	−5.08	−5.22	−4.9	−5.07	−3.1
CBM (eV)	−4.18	−3.85	−2.05	−3.6	−4	−3.9
Relative permittivity	10	10	3	3.5	3	3.5
Electron mobility (cm^2^/Vs)	100	5	2 × 10^−4^	1 × 10^−4^	2 × 10^−5^	1 × 10^−5^
Hole mobility (cm^2^/Vs)	25	2	2 × 10^−4^	2 × 10^−5^	1.6 × 10^−5^	1 × 10^−7^
CB effective DOS (cm^−3^)	2.5 × 10^18^	2.2 × 10^18^	2.2 × 10^18^	1 × 10^21^	1 × 10^21^	1 × 10^21^
VB effective DOS (cm^−3^)	1.9 × 10^19^	1.8 × 10^19^	1.8 × 10^19^	1 × 10^21^	1 × 10^21^	1 × 10^21^
Donor density (cm^−3^)	3.25 × 10^17^	-	-	-	-	-
Acceptor Density (cm^−3^)	-	1 × 10^15^	2 × 10^19^	1 × 10^19^	-	-

**Table 2:** A comparison between experimental and simulated PV metrics of organic and Sb_2_Se_3_-based solar cell devices. The experimental data of the organic cell has been extracted from REF [36], while that for the Sb_2_Se_3_ cell has been extracted from REF [38].

PV Metrics		*J*_sc_ (mA/cm^2^)	*V*_oc_ (V)	FF (%)	PCE (%)
Organic Cell	Exp. data	0.914 ± 0.010	14.36 ± 0.17	72.0 ± 1.0	9.45 ± 0.24
	Simulation	0.918	14.33	75	9.86
Sb_2_Se_3_ Cell	Exp. data	0.449	28.30	62.10	7.89
	Simulation	0.452	28.25	62.10	7.93

**Table 3:** p-i-n tandem factors for different transport layers demonstrating the enhancement relative percentage.

	*J*_sc_ (mA/cm^2^)	*V*_oc_ (V)	FF (%)	PCE (%)	Δ*ξ* (%)
Initial	13.61	1.38	60.21	11.28	0
Top HTL (CuI)	12.68	1.38	58.18	10.17	−9.84
Top ETL (ZnO)	13.89	1.72	79.85	19.05	68.88
Bottom HTL (CuI)	14.91	1.37	57.92	11.86	5.14
Bottom ETL (WO_3_)	13.64	1.37	60.04	11.19	−0.80
Enhanced with (ZnO & CuI)	15.02	1.71	79.51	20.45	81.29

**Table 4:** PV parameters of p-i-n tandem-, top, and bottom subcells under current matching point.

	*J*_sc_ (mA/cm^2^)	*V*_oc_ (V)	FF (%)	PCE (%)
Top cell	17.03	0.92	75.24	11.86
Bottom cell	17.03	0.76	74.83	9.70
Tandem cell	17.03	1.68	74.86	21.52

**Table 5:** n-i-p tandem factors for different transport layers demonstrating the enhancement relative percentage.

	*J*_sc_ (mA/cm^2^)	*V*_oc_ (V)	FF (%)	PCE (%)	Δ*ξ* (%)
Initial	14.32	1.36	74.65	14.50	0
Top ETL (ZnO)	14.27	1.36	74.59	14.43	−0.48
Top HTL (CuI)	13.53	1.37	76.93	14.27	−1.59
Bottom ETL (WO_3_)	14.30	1.63	75.61	17.57	21.17
Bottom HTL (CuI)	14.32	1.36	74.70	14.51	0.07
Enhanced with (WO_3_ & CuI)	14.30	1.63	75.63	17.58	21.24

**Table 6:** PV parameters of n-i-p tandem, top, and rear subcells under current matching condition.

	*J*_sc_ (mA/cm^2^)	*V*_oc_ (V)	FF (%)	PCE (%)
Top cell	15.73	0.92	75.08	10.88
Bottom cell	15.73	0.70	75.28	8.31
Tandem cell	15.73	1.62	74.92	19.14

**Table 7:** A comparison of PV performance parameters of various tandem configurations from experiment and simulation studies.

Front/Rear Subcells	*J*_sc_ (mA/cm^2^)	*V*_oc_ (V)	FF (%)	PCE (%)	REF.
Sb_2_S_3_/Si	18.04	1.64	82.41	24.34	[56]
Polymer/Si	16.43	2.04	84.81	28.41	[57]
Organic/Si (4T)	-	-	-	15.15	[58]
Sb_2_S_3_/Sb_2_Se_3_ (4T)	-	-	-	7.93	[28]
Sb_2_S_3_/Sb_2_(S_0_._7_Se_0.3_)_3_/Sb_2_Se_3_	11.08	3.44	86.49	32.98	[29]
PBDB-T:IDTTA/ PTB7-Th:IEICO-4F	13.10	1.68	68.00	14.70	[59]
p-i-n Organic /p-i-n Sb_2_Se_3_	17.03	1.68	74.86	21.52	This work
n-i-p Organic /n-i-p Sb_2_Se_3_	15.73	1.62	74.92	19.14	This work

## Data Availability

Not applicable.

## Supplementary Materials

The following supporting information can be downloaded at: https://www.mdpi.com/article/10.3390/polym15112578/s1, Figure S1: Contour graphs of p-i-n tandem efficiency dependency on the thickness of top and bottom absorber layers; Figure S2: Contour graphs of n-i-p tandem efficiency dependency on the thickness of top and bottom absorber layers; Table S1: Basic parameters of ZnO, WO_3_, and CuI transport layers.
