# Supplementary material for: Proposal and Numerical Analysis of Organic/Sb2Se3 All-Thin-Film Tandem Solar Cell
Source: Polymers (Basel). 2023 Jun 5;15(11):2578. doi: 10.3390/polym15112578 (PMC10255658; doi:10.3390/polym15112578)
Supplement: Supplementary file 1 [file polymers-15-02578-s001.zip › polymers-2420999-supplementary.pdf]

Supplementary Material

Proposal and Numerical Analysis of Organic/Sb_2Se_3 All-Thin-Film Tandem Solar Cell

Tarek I. Alanazi [1,*], Abdulaziz Alanazi [2], Ezzeddine Touti [2,3], Ahmed M. Agwa [2,4], Habib Kraiem [2,5], Mohana Alanazi [6], Abdulrahman M. Alanazi [2] and Mona El Sabbagh [7]

[1] Department of Physics, College of Science, Northern Border University, Arar 73222, Saudi Arabia
[2] Department of Electrical Engineering, College of Engineering, Northern Border University, Arar 73222, Saudi Arabia; af.alanazi@nbu.edu.sa (A.A.); esseddine.touti@nbu.edu.sa (E.T.); ahmad.agua@nbu.edu.sa (A.M.A.); habib.kraiem@yahoo.fr (H.K.); st201903004@stu.nbu.edu.sa (A.M.A.)
[3] Electrical Engineering Department, Laboratory of Industrial Systems Engineering and Renewable Energies (LISIER), University of Tunis, Tunis 1008, Tunisia
[4] Department of Electrical Engineering, Faculty of Engineering, Al-Azhar University, Cairo 11651, Egypt
[5] Processes, Energy, Environment and Electrical Systems, National Engineering School of Gabes, University of Gabes, Gabes 6029, Tunisia
[6] Department of Electrical Engineering, College of Engineering, Jouf University, Sakaka 72388, Saudi Arabia; msanazi@ju.edu.sa
[7] Engineering Physics and Mathematics Department, Faculty of Engineering, Ain Shams University, Cairo 11535, Egypt; mona.mohammed@eng.asu.edu.eg
* Correspondence: tarek.alanazi@nbu.edu.sa

Table S1. Basic parameters of ZnO, WO_3, and CuI transport layers.

**Parameters**	**ZnO [43]**	**WO_3\ [44]**	**CuI [53]**
Thickness (nm)	30	30	40
Energy gap (eV)	3.6	2.6	3.1
Electron affinity (eV)	4.1	3.8	2.1
Relative permittivity	8.5	4.8	6.5
Electron mobility (cm^2/Vs)	1	30	100
Hole mobility (cm^2/Vs)	0.1	30	43.9
CB effective density of states (cm^{-3})	7.3×10^{20}	2.2×10^{21}	2.8×10^{18}
VB effective density of states (cm^{-3})	2.8×10^{21}	2.2×10^{21}	1×10^{19}
Shallow donor density (cm^{-3})	5×10^{18}	5×10^{18}	-
Shallow acceptor density (cm^{-3})	-	-	5×10^{18}

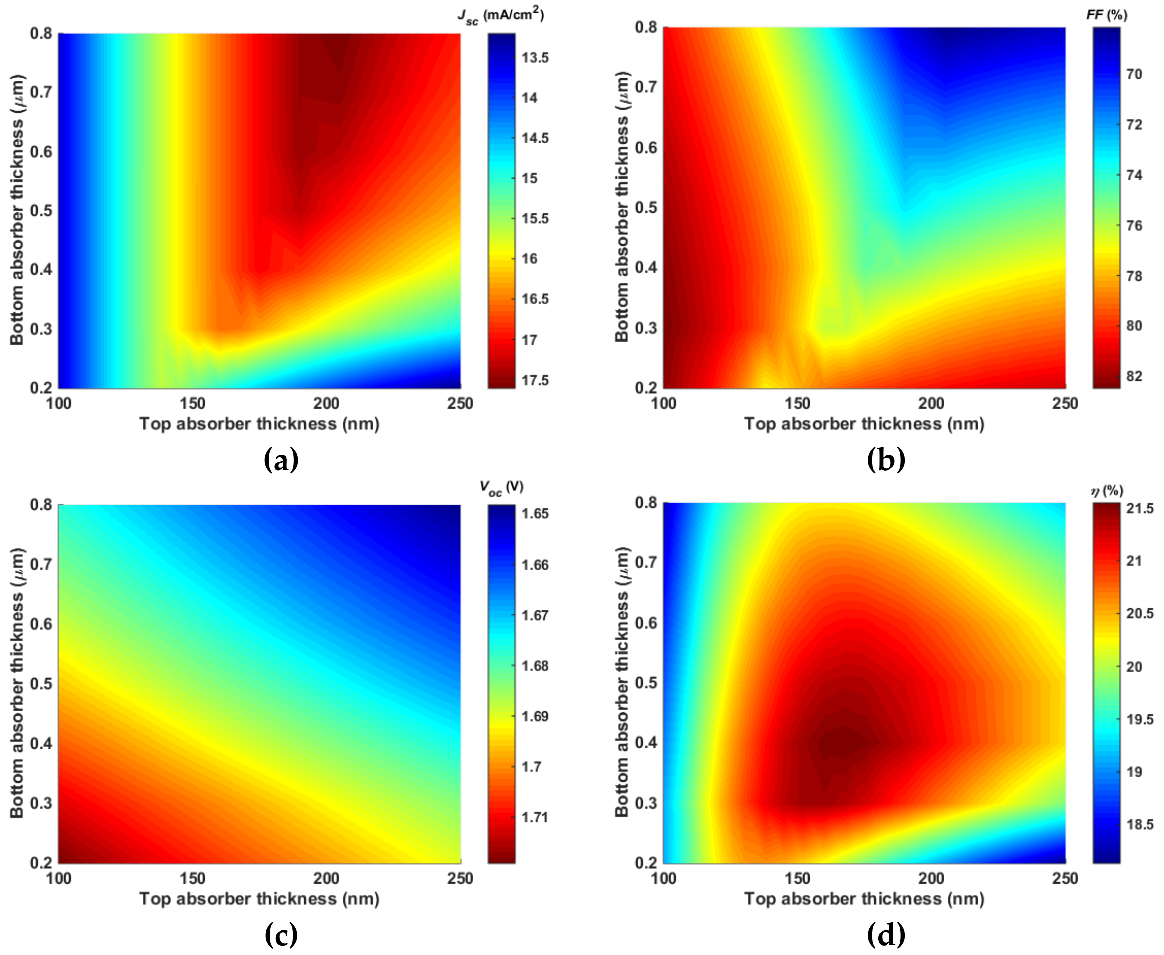

Figure S1. Contour graph of pin tandem efficiency dependency on the thickness of top and bottom absorber layers.

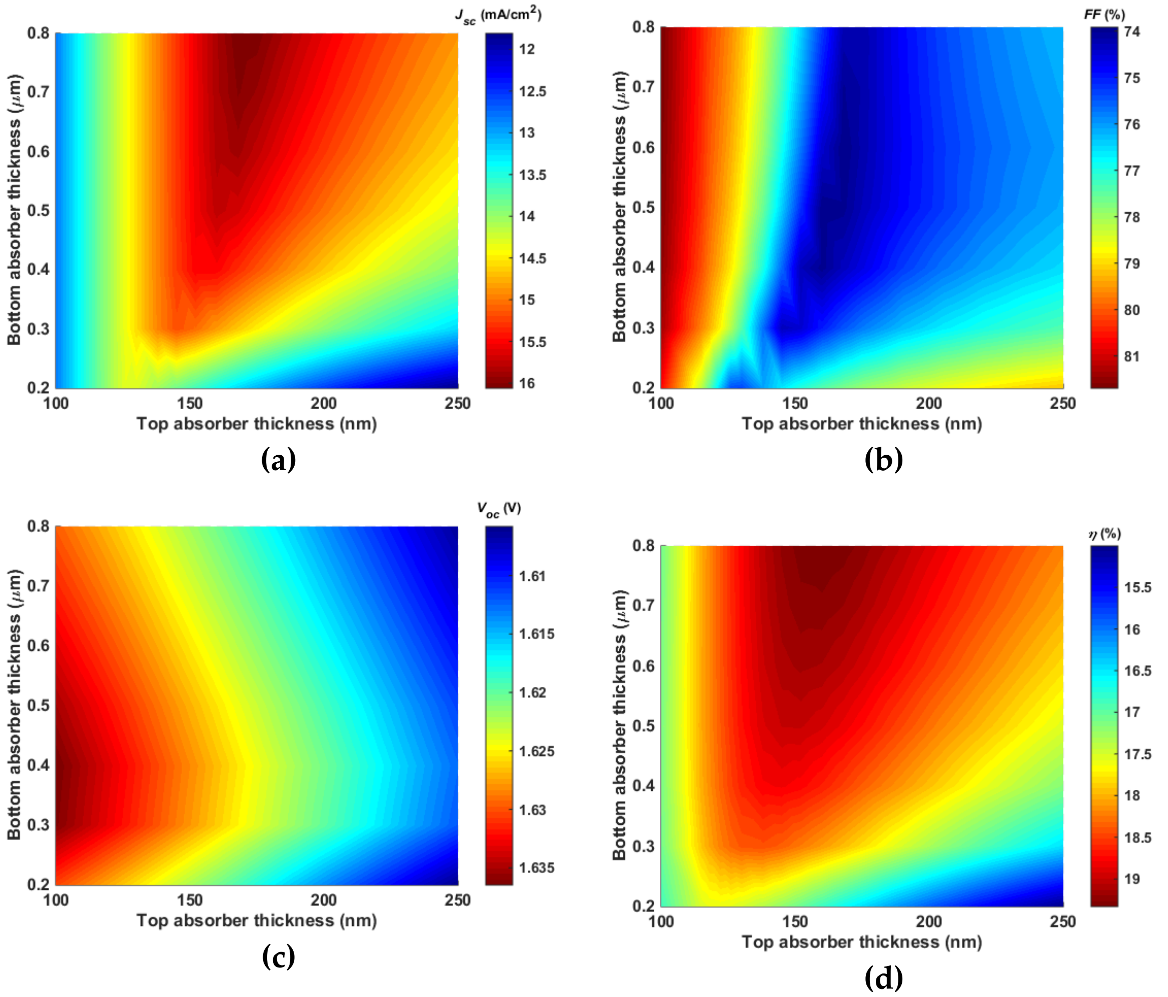

Figure S2. Contour graph of nip tandem efficiency dependency on the thickness of top and bottom absorber layers.
